# Substance-specific variability of ADHD symptoms in riga psychiatry and addiction medicine centre treatment-seeking substance use disorder outpatient population

**DOI:** 10.1192/j.eurpsy.2021.264

**Published:** 2021-08-13

**Authors:** M. Ennītis, M. Laizāne, N. Bezborodovs, I. Landsmane, S. Skaida

**Affiliations:** 1 Faculty Of Residency, Riga Stradins University, Riga, Latvia; 2 Department Of Psychiatry And Narcology, Riga Stradins University, riga, Latvia; 3 Department Of Addiction Medicine, Riga Psychiatry and Narcology Centre, Riga, Latvia; 4 Department Of Psychiatry And Narcology, VSIA Riga Center of Psychiatry and Narcology, Riga, Latvia

**Keywords:** ADHD, Substance Use Disorder, Adult Attention-Deficit / Hyperactivity Disorder, ASRS v.1.1

## Abstract

**Introduction:**

Studies show a link between attention deficit disorder (ADHD) and substance use disorders (SUD). Patients may abuse illicit drugs or alcohol as means of self-medicating e.g. stimulants for ADHD symptoms. Identifying ADHD symptoms in SUD patients could help improve treatment outcome and quality of life.

**Objectives:**

To evaluate the prevalence of ADHD symptoms in Riga Psychiatry and Addiction Medicine Centre (RPNC) outpatients and study the link between ADHD symptoms and specific SUDs.

**Methods:**

In a period of 30 days, all consentient clients of RPNC outpatient addiction clinic were surveyed for basic sociodemographic data and screened with Adult ADHD Self-report Scale (ASRSv1.1). Results were compared among patients with different types of addictions previously diagnosed using ICD-10 classification. Results were also compared to a control group.

**Results:**

Out of 279 participants, 209 were treatment-seeking SUD patients, 70 controls. Mean age was 37.99, 77.8% were male. Among SUD patients 45,2% had alcohol UD(F10), 52.9% opiate UD(F11), 35.1% stimulant UD(F14;f15), 20.2% sedative UD(F13). Patients who had stimulant addiction as one of their diagnosis and patients with multiple addictions were significantly more likely (p=0.023 and p=0.012 respectively) to screen positive for ASRSv1.1 among SUD patients population. All types of SUDs were significantly more likely to screen positive for ASRSv1.1 when compared to a control group.

**Conclusions:**

There is a strong link between SUD and ADHD symptoms. Patients with stimulant or multiple SUDs are more likely to screen positive for ADHD symptoms than other SUD patients. It is important to identify ADHD symptoms in treatment-seeking SUD patients.

**Disclosure:**

No significant relationships.

